# Anxiety and Depression in Tinnitus Patients: 5-Year Follow-Up Assessment after Completion of Habituation Therapy

**DOI:** 10.1155/2012/375460

**Published:** 2012-03-28

**Authors:** Eva-Signe Falkenberg, Ona Bø Wie

**Affiliations:** ^1^Department of Special Needs Education, Faculty of Educational Sciences, University of Oslo, P.O. Box 1140 Blindern, 0318 Oslo, Norway; ^2^Department of Otolaryngology, Oslo University Hospital, Rikshospitalet, P.O. Box 4950 Nydalen, 0424 Oslo, Norway

## Abstract

Treatment programs based on a neurophysiological model have shown a positive effect on anxiety and depression in tinnitus patients. The aim of this paper was to assess the *long-term effect* of tinnitus habituation therapy. Sixty-eight individuals were treated with a comprehensive therapy program. The degree of anxiety and depression was assessed before, after, and five years after intervention using the Hospital Anxiety and Depression Scale. The positive and significant changes achieved after habituation therapy (pre = 1.10, post = 0.92 for anxiety and pre = 0.77, post = 0.62 for depression) were maintained five years after treatment ended (0.87 for anxiety and 0.52 for depression). A regression analysis revealed that individual evaluation of the treatment lectures, self-reported health condition, individual experiences of hyperacusis, and hearing loss could explain 44.3% of the variation in anxiety and 30.5% of the variation in depression posttreatment. Five years after, individual evaluation of the treatment lectures and self-reported health condition explained 22.2% of the variation in anxiety. These factors and individual experiences of hyperacusis could further explain 34.9% of the variation in depression. The effect of a neurophysiologic-based management treatment was maintained five years after treatment ended, indicating that the patients continued the improvement process without becoming dependent on professionals.

## 1. Introduction

Tinnitus, defined as “the perception of sound that results exclusively from activity within the nervous system without any corresponding mechanical, vibratory activity within the cochlea, and not related to external stimulation of any kind” [1], occurs in 17% of the population [1, 2]. It is argued that approximately one third of the population experiences tinnitus at least once per lifetime, and approximately 1–5% develop serious psychosocial complications [3]. Tinnitus may be perceived in one or both ears or within the head and is experienced as continuous or intermittent. The sound varies from simple sounds, such as whistling, to complex sounds, such as music.

Tinnitus-related activity in the nervous system (TRA) is hypothesized to be present in every human being. The signal is usually a weak sound that does not cause suffering. It may be influenced by cochlear pathology. Tinnitus and hearing deficit are thus often related phenomena [3, 4]. Multifunctional connections between the auditory system, the limbic system, and the autonomic nervous system seem to be crucial in the development of tinnitus and tinnitus annoyance [5].

A close association between tinnitus and comorbid psychological disorders has been demonstrated [6–11], and a high prevalence of anxiety and depression has been reported in tinnitus sufferers [12–16]. It is also argued that tinnitus patients score lower on self-esteem and well-being assessments [16]. A screening or assessment for psychological distress in tinnitus sufferers is recommended to give the patients adequate treatment [11]. Researchers state that the consequences of chronic pain and tinnitus are similar: emotional effects, reduced involvement in work-related activities, interpersonal problems, and decreased opportunities to engage in previously enjoyable activities [15, 17, 18]. It is argued that tinnitus patients with no hearing loss tend to have more diagnoses per patient and more anxiety disorders than those with hearing loss [3, 19].

Jastreboff and Hazell argue that for most people, tinnitus perception is far from indicating any medical problem and is, in fact, a natural and benign experience that can be easily habituated. Only in rare cases is tinnitus caused by a medical problem (e.g., a vestibular schwannoma or acoustic tumor) that requires examination [20].

Nevertheless, for a considerable number of individuals, tinnitus is experienced as an annoying condition and a pressing reason for seeking professional help. An increasing number of patients have, over the last two decades, received treatment based on the neurophysiological model of understanding the process that leads to tinnitus and tinnitus annoyance [21]. The model is customarily labeled “the neurophysiological model of tinnitus” to distinguish it from other models involving neuroscience. As a crucial part of this model, it was proposed that inappropriate activation of the limbic and sympathetic part of the autonomic nervous systems by the tinnitus signal is responsible for behaviorally observed reactions to tinnitus, such as anxiety, problems with concentration, panic attacks, and suppression of the ability to enjoy activities in life [22]. Annoyance does not depend on the strength of the tinnitus-related activity but on the strength of the connection between the cerebral cortex, the auditory system, and the limbic and autonomic nervous system. The strength of this connection produces sufferers as opposed to nonsufferers [4]. The same type of reaction is observed after overstimulation of the limbic and autonomic nervous systems by many other factors, such as sleep deprivation, chronic pain, or sensory stimulation, which we cannot control [22]. A considerable number of tinnitus sufferers relate their first awareness of tinnitus to changes in their life, whether small or substantial. These changes could include such events as divorce, being laid off, sickness in the family, accidents, surgery, or even having had their ears syringed [4]. Such events can produce a change in the arousal of the brain or its homeostasis, resulting in a state of heightened brain arousal during which the tinnitus signal is registered cortically [23]. It is also likely that such events can weaken the normal mechanisms of defense against anxiety and other emotional reactions.

Based on the neurophysiological model presented by Jastreboff and Hazell, also denoted retraining therapy, habituation therapy, the three vital components in the therapeutic program are (1) *cognitive aspects*, such as belaboring fear and anxiety, (2) *relaxation*, and (3) *sound therapy*. The main goal of this therapy is to decrease the strength of the tinnitus signal within the brain (enhancing environmental sounds to effectively decrease the strength of the tinnitus signal and reduce activation of the limbic and autonomic nervous systems). This neurophysiological-based therapy means that the tinnitus-related neuronal activity is blocked from reaching the limbic and autonomic nervous systems, and, consequently, there are no negative reactions to tinnitus (habituation of reaction). Moreover, the auditory system is capable of blocking this tinnitus-related neuronal activity, preventing it from reaching higher cortical areas and thus being perceived (habituation of perception). Jastreboff and Hazell underline the importance of including all three components in a therapeutic program. Sound therapy without appropriate discussion and counseling based on neurophysiological processes does not work [20]. The concept “cognitive-behavioral treatment” when used in connection with the neurophysiological approach to tinnitus sometimes encompasses all three components mentioned above [8, 24, 25]. The theoretical background for the neurophysiological model is confirmed by Herraiz, who argues that progress in neuroscience research has given birth to new theories for tinnitus generation [26]. From the point of view in which cochlear dysfunctions would be considered the origin and maintenance mechanisms, the concept of the importance of compensation systems from the central auditory pathways has been introduced. These systems could act as the most relevant factor for chronic persistent tinnitus after peripheral aggression [26]. Several studies report positive long-term outcomes from treatments built on the neurophysiological model [4, 27–32]. The described results of these studies include cognitive and emotional aspects, such as anxiety and depression, as well as annoyance and influence on life quality. However, most of these studies are based on a follow-up assessment no longer than two years after completion of treatment. Only one research project is found to report a longer follow-up study of cognitive behavioral therapy, reporting stable improvements of the tinnitus parameters and stable additional symptoms 15 years after the completion of therapy [24].

That annoying tinnitus can cause several symptoms in tinnitus sufferers calls for a multidisciplinary approach to treatment. The neurophysiological model with its three components requires knowledge derived from the audiological field, the psychological field, and the field of relaxation therapy. Practitioners from the audiological field experience a need for more tinnitus knowledge among therapists representing other scientific fields.

The purpose of this paper is to describe the results of a long-term follow-up effect study that was performed five years after a completed tinnitus habituation therapy program based on Jastreboff and Hazell's neurophysiological model. The focus will be on anxiety and depression.

## 2. Materials and Methods

### 2.1. Participants

Eighty-three individuals participated in a one-year treatment program and completed a survey, the Hospital Depression and Anxiety Scale (HADS), both before and immediately after treatment. Out of these individuals, 68 (82%) completed the survey at the 5-year follow-up assessment. Of these 68, 32 (47%) were women and 36 were (53%) men. The age distribution for the group at the 5-year follow-up was 33–82 years with a mean age of 58.49. The results of the effect analyses were based on the assessment carried out immediately after the treatment ended were published in 2003 [4]. Before treatment, 19% of the participants had experienced tinnitus for less than one year, 10.3% had 1 to 2 years of experience, 20.6 had 2–5 years, 17.6 had 5–10 years and the largest group of 32.4% had experienced tinnitus annoyance for more than 10 years. Further demographic information is presented in Table 1.

### 2.2. The Treatment Program

The multidisciplinary team consisted of two medical doctors, two educational audiologists, a physiotherapist, and a psychologist. Due to the limitation of resources, the treatment was given in groups rather than individually. Each patient was invited to participate in eight so-called “main” group sessions. Three of these sessions were in the form of lectures with all participants present where a physician described the current knowledge of tinnitus generation, the process of hearing, and the aim and structure of rehabilitation. Each lecture session contained a section for questions and answers. A psychologist conducted two sessions. One session conducted by a physiotherapist was carried out in groups of 10–12 patients. Two counseling sessions were carried out by educational audiologists in groups of 8–10 patients. The fitting of a white noise generator or hearing aid was, however, carried out individually. A total of 20.6% of the participants were users of hearing aids, whereas 55% were daily users of a white noise generator (Viennatone AM/TI or Viennatone Silent Star). The rest used general environmental sound enrichment. The cognitive inputs and instructions to enable the participants to examine themselves were given systematically in the groups. The participants were also encouraged to share their experiences within the groups. Most of the patients participated in every session. To take account of individual needs, the patients were also offered extra sessions in smaller groups. A few of the patients were given individual counseling. The total program for each participant lasted for approximately one year. The treatment program carried out after a medical investigation included three main components: (1) systematic structured retraining aimed at making the tinnitus signal insignificant by taking away tinnitus-related misconceptions and emotions, such as fear and anger. This retraining was aimed at breaking the vicious circle of tinnitus perception at different levels. (2) Stress management training, including relaxation techniques, with the goal of understanding and reducing oversensitive reactions to perceived threats. This goal may be achieved by enhancing dormant central resources (e.g., improving general physical health), drawing on past skills and experiences, modifying attitudes and beliefs, considering emotional make-up, accepting appropriate social support, and improving the ability to relax. The goal of this component of the therapy is to adjust more readily and effectively to routine and major incidental life demands or changes. (3) The avoidance of silence by sound enrichment and sound therapy aimed at reducing the contrast between silence and tinnitus, for example, by using hearing instruments, white noise generators, or environmental sounds as appropriate in each individual case. The important notion here is that the patient habituates to these sounds of known origin, while at the same time reducing the awareness of the tinnitus signals, as they do not “stand out” in contrast to silence [4].

### 2.3. Design and Study Settings

This study employs a longitudinal survey design assessing the long-term effect of a tinnitus habituation program 5 years after the treatment ended. The participants were recruited from The Norwegian Association of the Hard of Hearing (HLF) and their School and Resource Center in Briskeby. The program was carried out at the University of Oslo, Department of Special Needs Education in cooperation with Briskeby. The follow-up investigation of the results was carried out at the University of Oslo.

### 2.4. Assessment Instruments

The collected data were based on The Hospital Anxiety and Depression Scale (HADS). HADS is a standardized 14-item self-rating questionnaire consisting of 4-step scales. The questionnaire was developed for use in general hospital outpatient departments as a screening test for emotional disorders, such as anxiety and depression [33]. The following indices were developed from this questionnaire. To assess the reliability of the indexes, we used Cronbach's alpha scale where 1.0 represents perfect reliability [34]. The anxiety index is based on the following statements: “I feel tense or “wound up”,” “I get a sort of frightened feeling as if something awful is about to happen,” “worrying thoughts go through my mind,” “I can sit at ease and feel relaxed,” “I get a sort of frightened feeling like “butterflies” in my stomach,” “I feel restless, as if I have to be on the move,” and “I get sudden feelings of panic.” The reliability of this index is 0.91. The depression index is based on the following statements: “I still enjoy the things I used to enjoy,” “I can laugh and see the funny side of things,” “I feel cheerful,” “I feel as if I have slowed down,” “I have lost interest in my appearance,” “I look forward with enjoyment to things,” and “I can enjoy a good book, listening to the radio or watching TV.” The reliability of this index is 0.85.

### 2.5. Analyses

Descriptive statistics were used to report the characteristics of the individuals. Pearson correlation coefficients were utilized to examine the relationships among the study variables. *T*-tests were used to compare the results within and between groups. An analysis of factors that may explain the informants' experience of anxiety and depression was performed incrementally. First, all of the relevant background variables were analyzed to reveal those that correlated significantly with either anxiety or depression. These selected variables were subsequently included in a multiple regression analysis with anxiety and depression as the dependent variables. The independent variables that did not contribute to an improved explanation of anxiety or depression in the multiple regression model were removed, and the model was recalculated. The analyses were performed using PASW 18. The level of significance was set to a *P* value of 0.05 or less (without correction for multiple testing).

### 2.6. Ethical Considerations

All of the patients signed a consent form. The Regional Committee for Medical Research Ethics (REK) and the Norwegian Social Science Data Services approved this project.

## 3. Results

The results obtained from the present study of the long-term effects five years after treatment ended show that the positive and significant effects on anxiety and depression, which were achieved immediately after tinnitus habituation therapy, were maintained. In fact, a considerable number of patients continued to improve. No significant difference in outcome was shown between the participants who had experienced tinnitus annoyance for more than 10 years and those who had experienced the annoyance for a shorter period of time.

### 3.1. Anxiety

Table 2 shows that the reduction of anxiety as reported immediately after treatment was 0.18 points (from 1.10 to 0.92) with a further slight reduction from posttreatment to five years after treatment assessment (from 0.92 to 0.87). The maximum score possible for anxiety was 3. The total reduction from pretreatment to five years after treatment assessment was 0.23 and, therefore, strongly significant (*P* = 0.002).

Table 3 shows the results form a simple bivariate correlation between the explored variables that correlated significantly with the degree of anxiety. For both posttreatment and at the 5-year follow-up, the strongest correlation was found between the participants' positive experience of the lecture given in the treatment and a better outcome of the treatment (0.463, *P* = 0.000, 0.353, *P* = 0.004, resp.). In addition, the self-reported pretreatment health condition correlated significantly both immediately after treatment and at the five-year follow-up.

The results from the multiple regression analysis on experienced anxiety are presented in Table 4. The analysis gave a coefficient of determination *R* ^2^ of  .227, indicating that the included variables were associated with 22.7% of the total variance in the experienced anxiety of people with tinnitus. The adjusted coefficient of determination *R*_adj_^2^ was 0.202. Only two variables showed significant effect. Those who experienced decreased anxiety in relation to tinnitus were those who stated post-treatment that they were most satisfied with the lectures in the treatment and those who had the best self-reported health condition before treatment started. The size of each standardized regression coefficient showed that the experienced effect of the lectures explained most of the variation in anxiety, when the other variable was kept constant.

### 3.2. Depression

Table 2 shows a reduction of depression immediately after treatment of 0.15 points (from 0.77 to 0.62). The depression score five years after treatment shows a further slight reduction of 0.07 points (from 0.62 to 0.55). The maximum score possible for depression was 3. From the pretest to the assessment five years after treatment, the reduction was 0.22 points (*P* = 0.000).

Table 5 shows the results form a simple bivariate correlation between the explored variables that correlated significantly with the degree of depression. For both post-treatment and at the 5-year follow-up, the strongest correlation was found between the participants' self-reported pre-treatment health condition and improved outcomes of the treatment (0.378, *P* = 0.002, 0.411, *P* = 0.001, resp.). The analysis shows that the same variables that were of significant importance right after the treatment were also significant at the 5-year follow-up time point.

The results from the multiple regression analysis over the variation in experienced depression are presented in Table 6. The analysis gave an *R* ^2^ of 0.349, indicating that the included variables were associated with 34.9% of the total variance in the experienced anxiety (the *R*_adj_^2^ was 0.305). Reduced depression in relation to tinnitus was associated with the feeling of being in good health pre-treatment, the absence of hyperacusis, and high satisfaction with the lectures given as part of the intervention. The size of each standardized regression coefficient shows that the pre-treatment health condition and the absence of hyperacusis equally explained most of the variation in depression, when the other variable was kept constant.

## 4. Discussion

In line with the results of other studies [4, 6, 11], we found a higher prevalence of anxiety than depression. The participants further reported that they experienced significantly less anxiety and depression five years after neurophysiologically based tinnitus treatment than before treatment, indicating a long-term effect of such therapy. The findings confirm results from other studies based on shorter follow-up times [4, 27–32], suggesting that modified tinnitus retraining therapy may lead to subjective improvements in tinnitus-related symptoms, such as emotional and cognitive distress and intrusiveness. The results are also in line with those of Goebel et al. [24], who, in a 15-year prospective comparable follow-up study, reported improvements of the tinnitus parameters and additional symptoms. This result indicates that the positive findings in the present study, which were based on a five-year follow-up study, will also be maintained at a later point in time.

Our findings show a strong effect of the treatment related to the positive experience of the treatment lectures. The significant correlation between the participants' positive experience of the educational, medical, psychological, and physiotherapeutic lectures indicate that all of these aspects are of importance in the treatment offered. This result indicates the crucial importance of information that helps the patients understand the neurophysiological model of tinnitus and the importance of motivation for practicing the advice given to them during the treatment period.

Self-reported good health was shown to be a positive indicator for outcome, which could indicate that individuals reporting good health before treatment are more likely to benefit more from this kind of treatment than individuals reporting a poor health condition.

Further data indicate that the patients who suffer from both tinnitus and hyperacusis are those with the lowest therapy outcome. Our finding may support the suggestion of Jastreboff and Hazell [20] that in these individuals, one must start treating hyperacusis first.

The post-treatment results show that the habituation treatment outcome regarding anxiety was higher in the individuals with pre-treatment hearing impairment compared with those with no hearing loss. Other studies suggest that subjective discomfort is more present in tinnitus patients with normal hearing than in those with hearing impairment. Our findings obtained five years after treatment, however, did not show a significant effect of pre-treatment hearing status and the degree of anxiety.

Fifty-two percent of the participants in our study reported their first awareness of tinnitus to be related to a change in their life. Being aware of this common experience and the possible consequences for tinnitus annoyance can be a crucial step in the habituation process. The documentation of a close relationship between tinnitus and comorbid psychological disorders must also be taken into account in the initial phase of treatment [11]. Adoga et al. stress this point by recommending screening or assessment for psychological distress in tinnitus sufferers such that patients can be adequately treated [11].

### 4.1. Study Limitations

The placebo effect, defined as “a temporary (two to three months) improvement of symptoms not related to the effect of the treatment” with tinnitus ranges around 40%. Therefore, the potential impact of a placebo effect on the results should be examined carefully [20]. However, Jastreboff and Hazell argue that a sustained result over 6–24 months cannot be a placebo effect [20]. Habituation therapy in the present study shows a significant long-term effect of 5 years, and the importance of the placebo effect can thus be reduced or eliminated.

A control group for the current study would have been desirable; such a group was, however, not applicable for several reasons, mainly ethical aspects. It was not ethically acceptable to allow a group of patients to be let out of treatment for a period of five years.

## 5. Conclusions

Neurophysiologically based management treatment showed a longitudinal positive effect on anxiety and depression in tinnitus suffers five years after completed treatment. The results indicate that the participants have learned how to cope with their tinnitus annoyance and how to continue their improvement after completing therapy without becoming dependent on professionals.

## Figures and Tables

**Table 1:** Demographic information about the study sample (*N* = 68).

	% Yes	% No
Others in the family with tinnitus annoyance	36.9%	63.1%
Live alone	32.4%	67.6%
Working	54.4%	45.6%
Pretreatment hearing status (Audiogram)	69.0%	31.0%
Use of hearing aids	20.6%	79.4%
Daily use of sound generator	55.0%	45.0%
Hyperacusis	34.0%	66.0%
Tinnitus awareness related to changes in life	52.2%	47.8%

**Table 2:** Mean scores and standard deviation regarding anxiety and depression at pre- and posttreatment and after 5 years of follow-up (*n* = 68).

	Pre-treat	Post-treat	Five-year follow-up	Mean difference pretest and posttest	Mean difference pretest and 5-years follow-up
Anxiety	1.10 (0.64)	0.92 (0.55)	0.87 (0.64)	0.18 (*P* = 0.001)	0.23 (*P* = 0.002)
Depression	0.77 (0.56)	0.62 (0.52)	0.55 (0.47)	0.15 (*P* = 0.002)	0.26 (*P* = 0.000)

**Table 3:** Results from bivariate correlation analyses over factors associated with the degree of tinnitus-related anxiety.

	*N*	Anxiety posttreatment	Anxiety five-year follow-up
Experienced effect of lectures	65	.463**	.353**
Self-reported health pretreatment	67	.316**	.351**
Hyperacusis	50	.296*	.130
Pretreatment hearing status	58	−.262*	−.146

**Correlation is significant at the 0.01 level (2-tailed).

*Correlation is significant at the 0.05 level (2-tailed).

**Table 4:** Results of simultaneous multiple regression of the variation in anxiety after 5-year follow-up (*n* = 65).

	Correlations		Standardized coefficients	Multiple correlation
Independent variable	Dependent variable Anxiety 5-year follow-up	Independent variable Experienced effect of lectures	Beta	

Experienced effect of lectures	.353**		. 324**	
				*R*² = .227**
Self-reported pretreatment health	.351**	.093	.321**	
			

Adjusted *R*² = .202**.

**Correlation is significant at the 0.01 level (2-tailed).

*Correlation is significant at the 0.05 level (2-tailed).

**Table 5:** Results from bivariate correlation analyses over factors associated with the degree of tinnitus-related depression.

	*N*	Depression post-treatment	Depression after five years of follow-up
Self-reported pre-treatment health	67	.379**	.411**
Hyperacusis	50	.337*	.396**
Self-reported post-treatment health	66	.337**	.363**
Experienced effect of lectures	65	.321**	.294*

**Correlation is significant at the 0.01 level (2-tailed).

*Correlation is significant at the 0.05 level (2-tailed).

**Table 6:** Results of simultaneous multiple regression of the variation in depression after 5-year follow-up (*n* = 45).

	Correlations			Standardized coefficients	Multiple correlation
Independent variable	Dependent variable	Independent variable			

	Depression post treatment	Self-reported pre-treatment health	Pre-treatment hyperacusis	Beta	

Self-reported pre-treatment health	.369**			.342**	
Pre-treatment hyperacusis	.411**	.132		.342**	*R*² = .349**
Experienced effect of lectures	.294**	.093	.036	.249*	

Adjusted *R*² = .305**.

**Correlation is significant at the 0.01 level (2-tailed).

*Correlation is significant at the 0.05 level (2-tailed).
